# A surgical technique for secondary repair of obstetric anal sphincter injuries; sphinctero-vagino-perineoplasty

**DOI:** 10.4274/tjod.12369

**Published:** 2019-01-09

**Authors:** Arife Şimsek, Mustafa Ateş, Abuzer Dirican, Dinçer Özgör

**Affiliations:** 1 İnönü University Faculty of Medicine, Department of General Surgery, Malatya, Turkey

**Keywords:** Anal incontinence, obstetric anal sphincter injury, perineoplasty, sphincteroplasty, vaginoplasty

## Abstract

**Objective:**

This study was conducted to present the preliminary results of seven patients treated with sphinctero-vagino-perineoplasty for secondary repair of obstetric anal sphincter injuries.

**Materials and Methods:**

This retrospective study was conducted on the records of seven patients who underwent secondary repair of obstetric anal sphincter injuries at the colorectal surgery unit of a tertiary care center between February 2015 and December 2017.

**Results:**

All patients with solid stool incontinence were fully recovered at postoperative month 3. The Wexner incontinence score was significantly improved (decreased from 14.12 [range: 8-20] to 2.28 [range: 1-4]). The complication rate was 85.7% (wound infection, abscess, hematoma, detachment).

**Conclusion:**

Combined repair of anal sphinchters, perineal body, superficial transverse perineal muscles, and bulbospongious muscles, which contribute to anal continence, may improve surgical outcomes in patients with obstetric anal sphincter injuries.

**PRECIS:** We present the preliminary results of seven patients treated with sphinctero-vagino-perineoplasty for secondary repair of OASIS.

## Introduction

The most common cause of anal incontinence in women is obstetric anal sphincter injuries (OASIS). *Clinically apparent* OASIS occur in less than 3 percent of vaginal deliveries^(1)^. In spite of primary repair, up to 5% of these patients develop anal incontinence, which severely impairs their quality of life^(2,3)^. When OASIS is treated for anal incontinence, it is considered as a secondary repair even if no primary repair has been performed during the postpartum period^(^^4)^. There is no consensus on which surgical technique is effective for secondary repair of OASIS^(5)^. Unfortunately, long-term consequences of surgical interventions (sphincteroplasty, perineorrhaphy, and transposition of muscle flaps) are unsatisfactory. All of these techniques are focused on either isolated anal sphincter repair, or reconstruction of tissue supporting the anal canal with the anal sphincter unrepaired. We believe that the damage to the perineal body, superficial transverse perineal muscles, and bulbospongiosus muscles, which provide contribution to anal continence, is ignored, especially in direct surgical repair. Therefore, the success rates of conventional techniques focused on anal sphincter repair for OASIS are low. In this study, we present the preliminary results of a surgical technique that involves combined restoration of vaginal, perineal, and anal sphincter muscles, for secondary repair of OASIS.

## Materials and Methods

The medical records of 7 women who underwent secondary repair of OASIS at a colorectal surgery unit of a tertiary care center between February 2015 and December 2017 were reviewed. Prior to the surgery, endoanal ultrasonography (EAUS) was performed to measure the degree of sphincter defect. Wexner incontinence scores (WIS) were recorded in both the preoperative and postoperative periods. Postoperative complications and follow-up periods were also recorded. The menstrual cycle was taken into account. Progesterone supplements were given to delay menstruation in the postoperative period. Sexual intercourse was prohibited for 3 months postoperatively.

### Surgical technique

**Figure 1:**
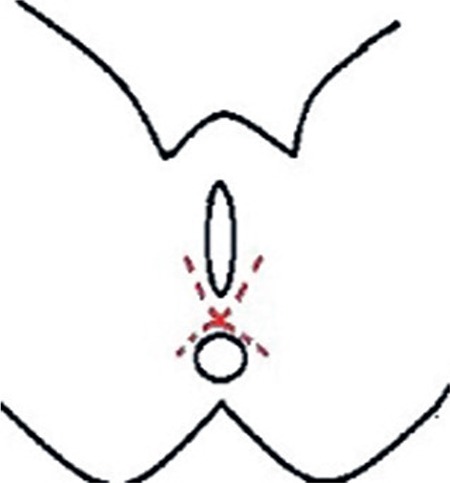
A semicircular incision matched the projection of the bulbospongiosus muscles

**Figure 2:**
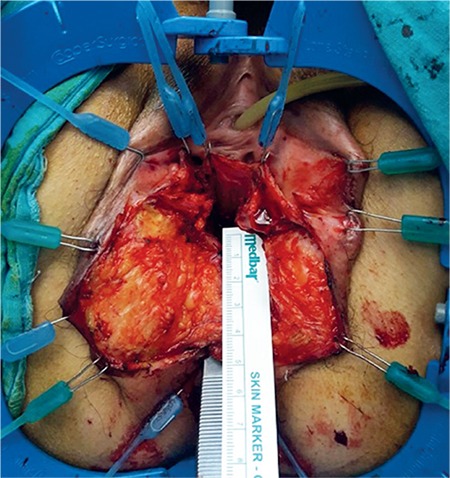
The vaginal mucosa was dissected at a depth of 6 cm, extending laterally to the bulbospongiosus and puborectal muscles and inferiorly to the perineal body. The anorectal mucosa was dissected from the sphincter muscles at a depth of 5 cm. The anal sphincters were dissected free with at least a depth of 4 cm

**Figure 3:**
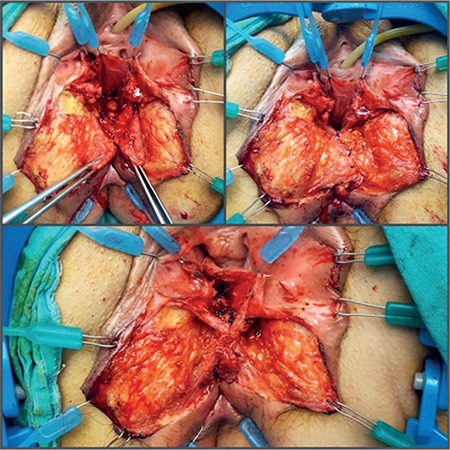
The retracted ends of the sphincters were repaired using the overlapping method. The perineal body was formed on EAS and the anus was centralized through end-to-end repair of the free ends of the bulbospongiosus muscles.

**Figure 4:**
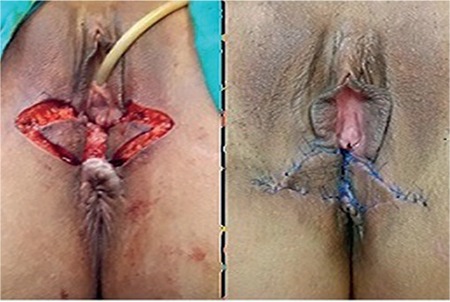
V-Y advancement flap between the vagina and the anus

All patients underwent surgery in the lithotomy position under spinal anesthesia. First, a semicircular incision was made matching the projection of the anal sphincter. The second U-shaped incision was made on the posterior commissure of the bulbospongiosus muscles (Figure 1). The vaginal mucosa was dissected to a depth of 6 cm, extending laterally to the bulbospongiosus and puborectal muscles and inferiorly to the perineal body. The anorectal mucosa was dissected from the sphincter muscles at a depth of 5 cm. The anal sphincters were dissected free with at least a depth of 4 cm (Figure 2). The retracted ends of the sphincters were identified and repaired using the overlapping method using 3/0 polydioxanone sutures. The perineal body was formed on the external anal sphincter (EAS) and the anus was centralized by end-to-end repair of the free ends of the bulbospongiosus muscles (Figure 3). A subcutaneous Penrose drain was placed. Anoderm, vagina, and perineal skin were sutured with absorbable materials. V-Y advancement flaps were used in patients with tissue defects between the vagina and anus (Figure 4). Protective ileostomy was performed only in cases where tissue loss of the anal canal and vagina (cloaca-like deformity) were noticed.

### Statistical Analysis

Statistical Package for the Social Sciences 17.0 for Windows Data was used. Descriptive frequencies were applied.

This study was conducted according to the principles of the 1975 Helsinki Declaration, which was revised in 2000. Informed consent was obtained from patients.

The study was approved by the İnönü University Local Ethics Committee (approval number: 2017/12-6). Informed consent forms were completed by all participants.

## Results

The average age of the women was 34.85 (range, 23-42) years, the average parturition number was 3.14 (range, 1-7), and the mean body mass index (BMI) was 27.14 (range, 22.7-30.4) kg/m^2^. All patients had a history of vaginal delivery. They developed symptoms of anal incontinence immediately after their first vaginal birth, and gradually worsened in the following births. The mean duration of symptoms was 39.4 (range, 6-120) months. EAUS showed both complete defect of EAS and complete and/or partial defect of the internal anal sphincter (IAS) in 6 patients. There was EAS defect only in one patient. The anal sphincter defect angle did not exceed 130 degrees in any patient. One patient reported incontinence to gas and liquid, and six patients reported incontinence to solid stool, liquid, and gas (Table 1). All six patients with solid stool incontinence were fully recovered at postoperative month 3. The mean preoperative WIS was 14.42 (range, 8-20), whereas it was 2.28 (range, 1-4) at postoperative month 3. The postoperative complications (wound infection, abscess, detachment, hematoma) developed in 6 patients (85.7%). V-Y advancement flaps were used in two of these patients, and free skin flap transfer was performed in one patient. The average follow-up duration was 12.28 (range, 3-26) months.

## Discussion

There are no randomized controlled trials comparing primary and secondary repair of OASIS due to ethical obstacles^(6)^. Based on observational studies, results of primary repair, especially performed by experienced surgeons, are superior to secondary repair^(7,8)^. Direct techniques include reconstruction of the sphincter itself, either by end-to-end or overlapping methods^(9,10)^. Indirect techniques include reconstruction of striated muscles or fasciae surrounding anal canal, and transposition of a striated muscle flap^(11,12,13)^. In the literature, surgical techniques are focused primarily on isolated anal sphincter repair^(9,10,14,15)^. Although short-term results of sphincteroplasty are satisfactory with 75-86% improvement in incontinence, they attenuate with time notifying that less than 50 percent of patients are still continent after 5-10 years^(16)^. The retraction of sphincters and overlooked pudendal injury may predispose to failure in sphincteroplasty^(15,17,18)^. Indirect methods in which anal sphincters remain unrepaired have poor functional results. The muscles supporting the anal canal necessitate conscious voluntary effort, so it is difficult to maintain continence for prolonged periods, and impossible during sleep^(17)^. As a result, the success rates of conventional surgical techniques decrease with time after surgery^(19,20,21)^. All of these techniques are focused on either isolated anal sphincter repair, or reconstruction of tissue supporting the anal canal without anal sphincter repair. We believe that the damage to the perineal body, superficial transverse perineal muscles, and bulbosupongiosus muscles, which provide contribution to anal continence, is ignored, especially in direct surgical repair. In the present study, all patients with solid stool incontinence were fully recovered at postoperative month 3. The improvement in WIS was statistically significant. Although complications were high they were resolved properly. We performed diverting colostomy in only two patients because diverting colostomy was not obligatory in the treatment of OASIS. Venkatesh et al.^(22)^ also reported favorable results of combined surgery in 44 patients with traumatic cloaca, the majority of which were secondary to obstetric injuries. They used puborectalis interposition, sphincteroplasty and perineal body repair. All patients except five, had regained both fecal and gas continence. Five women also improved following biofeedback therapy. Anaraki et al.^(23)^ performed sphincteroplasty and perineoplasty with skin advancement flap to reform the perineal body in 19 women with traumatic cloacal defects. *Significant improvement *in FI scores (decreased from 12.7 to 2.6)*, quality of life* (increased from mean of 45 to 95), dyspareunia (decreased from mean of 5 to 0.8) and sexual function satisfaction (increased from mean score of 0.2 to 4.7) in these patients *encouraged them to *recommend this technique as an effective surgical method. Their complication rate was 15.7% (wound infection in 2 patients, rectovaginal fistula in one patient), which was managed conservatively. In the current study, although the FI score was significantly improved (decreased from 14.12 to 2.28), the complication rate was higher (85.7%).

## Conclusion

Although the small sample size and absence of long-term results were limitations of this study, the satisfactory preliminary results encourage us to consider that combined repair of anal sphincters, perineal body, superficial transverse perinei muscles, and bulbospongiosus muscles may improve surgical outcomes in patients with OASIS.

## Figures and Tables

**Table 1:**
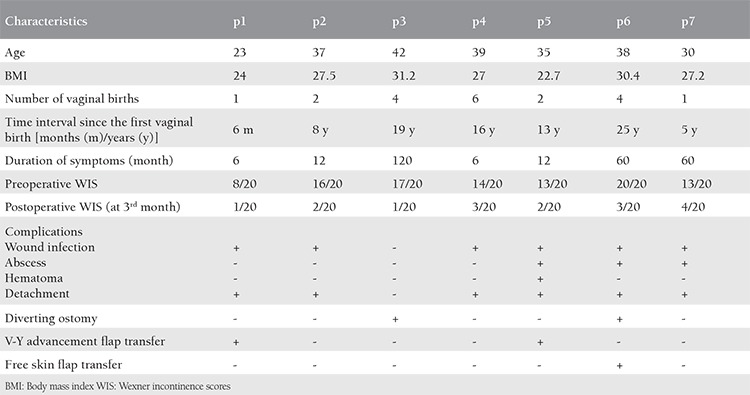
The characteristics of patients

## References

[1] Zetterstrom JP, Lopez A, Anzen B, Dolk A, Norman M, Mellgren A (1999). Anal incontinence after vaginal delivery: a prospective study in primiparous women. British Journal of Obstetrics and Gynaecology.

[2] Grigoriadis T, Mylona SC, Giannoulis G, Athanasiou S, Antsaklis A (2015). Sonographic Evaluation of Obstetric Anal Injuries. Donald School Journal of Ultrasound in Obstetrics and Gynecology.

[3] Fitzpatrick M, Fynes M, Cassidy M, Behan M, O’Connell PR (2000). Prospective study of the influence of parity and operative technique on the outcome of primary anal sphincter repair following obstetrical injury. Eur J Obstet Gynecol Reprod Biol.

[4] Sultan AH (2003.). Primary and Secondary Anal Sphincter Repair. In: Stanton S.L., Zimmern P.E. (eds) Female Pelvic Reconstructive Surgery. Springer.

[5] Fernando RJ, Sultan AH, Radley S, Jones PW, Johanson RB (2002). Management of obstetric anal sphincter injury: a systematic review & national practice survey. BMC Health Serv Res..

[6] Edozien L (2005). Primary versus secondary repair of obstetric anal sphincter injury. Clinical Risk.

[7] Sultan AH, Kamm MA, Hudson CN (1995). Obstetric perineal tears: an audit of training. J Obstet Gynaecol.

[8] Schofield PF, Grace R (1999). Faecal incontinence after childbirth. Clinical Risk.

[9] Besnard C, Bohec C, Dehni N, Collet M, Homer L (2009). How I do. . . the management of fourth degree perineal injury following vaginal delivery?. Gynecol Obstet Fertil.

[10] Zutshi M, Tracey TH, Bast J, Halverson A, Na J (2009). Ten-year outcome after anal sphincter repair for fecal incontinence. Dis Colon Rectum..

[11] Felt-Bersma RJ, Cuesta MA (1994). Fecal incontinence 1994: which test and which treatment?. Neth J Med.

[12] Whitehead WE, Wald A, Norton NJ (2001). Treatment options for fecal incontinence. Dis Colon Rectum..

[13] Conzo G, Brancaccio U, Esposito MG, Miranda G, Palazzo A, Stanzione F, Celsi S, Livrea A (2006). Surgical treatment of fecal incontinence secondary to obstetric trauma. Ann Ital Chir..

[14] Johnson E, Carlsen E, Steen TB, Backer Hjorthaug JO, Eriksen MT, Johannessen HO (2010). Short- and long-term results of secondary anterior sphincteroplasty in 33 patients with obstetric injury. Acta Obstet Gynecol Scand..

[15] Rothbarth J, Bemelman WA, Meijerink WJ, Buyze-Westerweel ME, van Dijk JG, Delemarre JB (2000). Long-term results of anterior anal sphincter repair for fecal incontinence due to obstetric injury/with invited commentaries. Dig Surg.

[16] Saldana Ruiz N, Kaiser AM (2017). Fecal incontinence - Challenges and solutions. World Journal of Gastroenterology..

[17] Browning GG, Motson RW (1984). Anal sphincter injury. Management and results of Parks sphincter repair. Annals of Surgery.

[18] Neill ME, Parks AG, Swash M (1981). Physiological studies of the anal sphichter musculature in fecal incontinence and rectal prolapse. Br J Surg.

[19] Fitzpatrick M, O’Herlihy C (2005). Short-term and long-term effects of obstetric anal sphincter injury and their management. Curr Opin Obstet Gynecol.

[20] Dudding TC, Vaizey CJ, Kamm MA (2008). Obstetric anal sphinchter injury: incidence, risk factors, and management. Ann Surg.

[21] Zetterström J, López A, Anzén B, Norman M, Holmström B, Mellgren A (1999). Anal sphincter tears at vaginal delivery: risk factors and clinical outcome of primary repair. Obstet Gynecol..

[22] Venkatesh KS, Ramanujam P (1996). Surgical treatment of traumatic cloaca. Dis Colon Rectum.

[23] Anaraki Fakhrolsadat, Etemad Omid (2017). Sphincteroplasty and perineoplasty with skin advancement flap in management of traumatic cloacal defect. J. Coloproctol. (Rio J.).

